# Supplementary material for: Improving resolution in quantum subnanometre-gap tip-enhanced Raman nanoimaging
Source: Sci Rep. 2016 May 25;6:25788. doi: 10.1038/srep25788 (PMC4879532; doi:10.1038/srep25788)
Supplement: Supplementary Information [file srep25788-s1.pdf]

Supplementary Information for

Improving resolution in quantum subnanometre-gap tip-enhanced Raman nanoimaging

Yingchao Zhang[1,2], Dmitri V. Voronine[1,3*], Shangran Qiu[1,2], Alexander M. Sinyukov[1], Mary Hamilton[3], Zachary Liege[1,3], Alexei V. Sokolov[1,3], Zhenrong Zhang[3] and Marlan O. Scully[1,3,4]

[1]*Texas A&M University, College Station, TX 77843, USA*

[2]*Xi'an Jiaotong University, Xi'an, Shaanxi 710049, China*

[3]*Baylor University, Waco, TX 76798, USA*

[4]*Princeton University, Princeton, New Jersey 08544, USA*

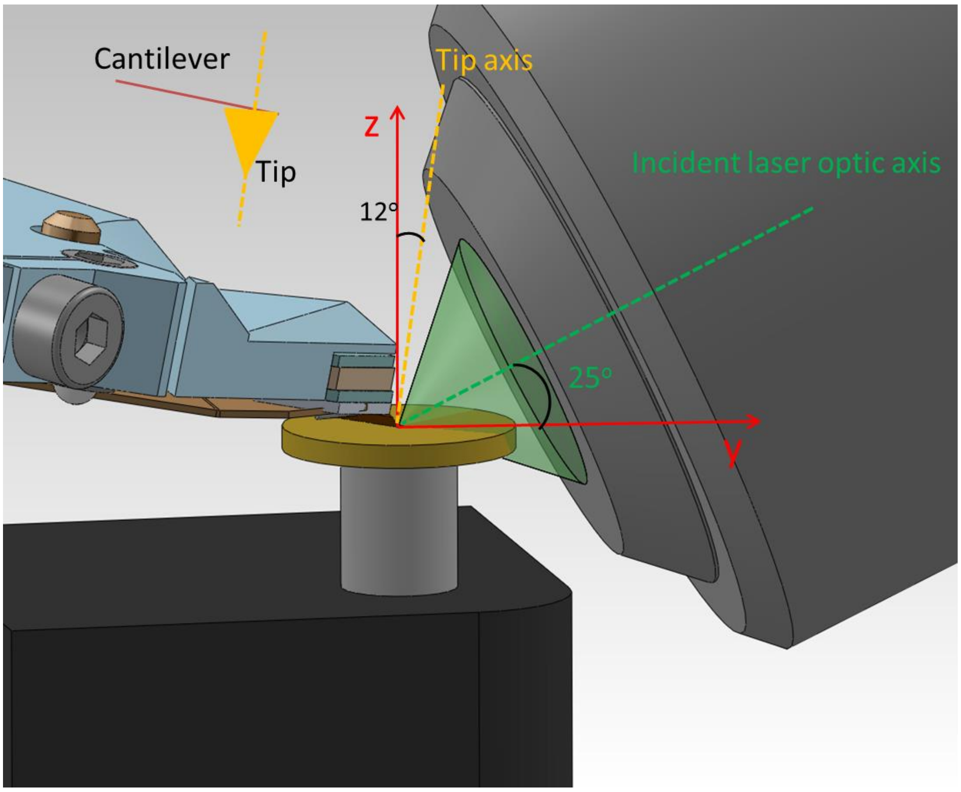

Supplementary Figure 1. Schematic of the tip-enhanced Raman scattering (TERS) experiments using a combination of scanning probe microscope (OmegaScope-R, AIST-NT) and Raman microscope (LabRAM HR Evolution, Horiba). 660 nm laser (green) is focused on the gold tip. The angle between the incident laser optical axis and y axis is 25°. The angle between the tip axis and z axis is 12°. The frame of axes is the same as shown in Fig.1a. The scattered Raman signal is collected by the illumination objective. The tip and the laser are stationary, while the sample is scanned in x, y, and z-directions.

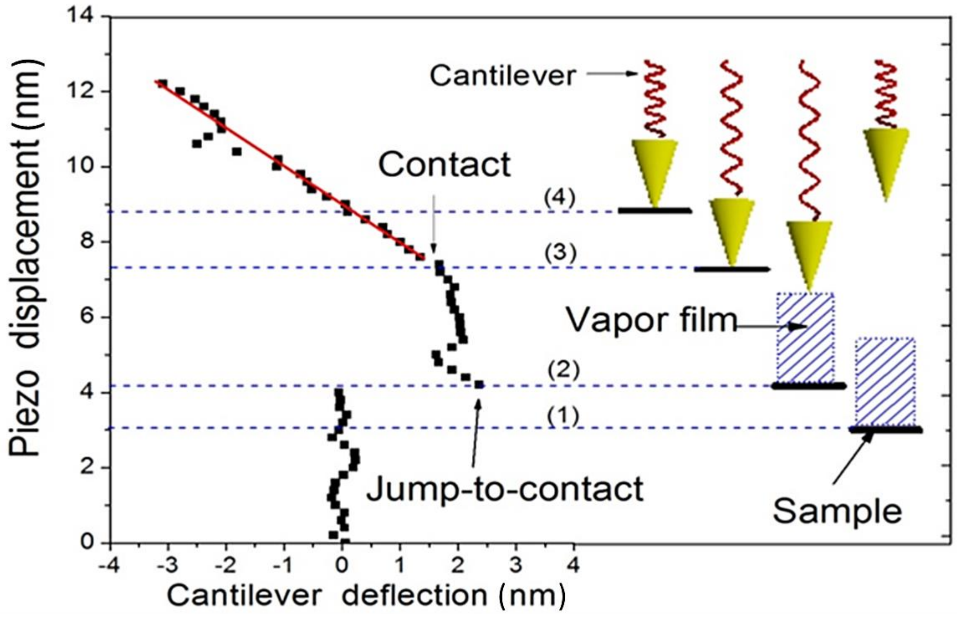

Supplementary Figure 2. Displacement-deflection curve. The cantilever deflection is plotted vs the tip-sample displacement of the AFM piezo actuator. The red solid straight line shows that after the tip-sample contact the cantilever is deflected linearly, as the tip and the sample move upwards together. The corresponding tip and sample states at four typical moments are highlighted by dashed lines.

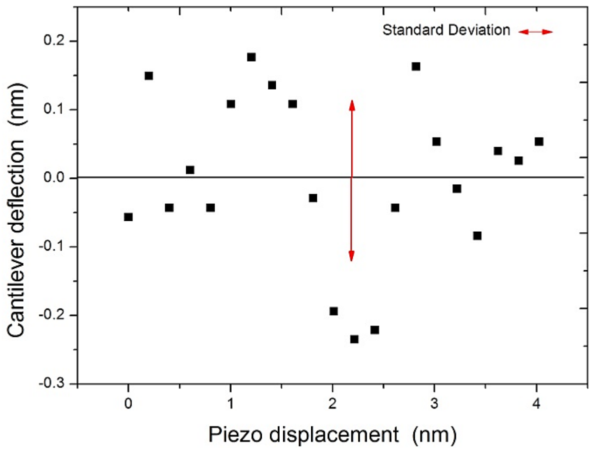

Supplementary Figure 3. Deviation of tip-sample distance before vdW contact. Before vdW contact, the tip-sample distance deviation results from both cantilever and piezo displacements. Fluctuation of the cantilever deflection before vdW contact has 0.12 nm standard deviation. The piezo displacement has the inherent deviation ~ 0.1 nm. Thus, the total deviation of the tip-sample distance before vdW contact is $\sqrt{0.12^2 + 0.1^2} \approx 0.16$ nm.

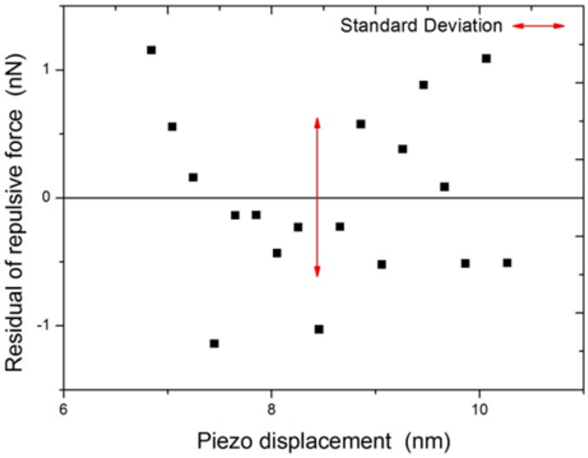

Supplementary Figure 4. Deviation of tip-sample distance after vdW contact. After vdW contact, the deviation of the tip-sample distance is mainly due to the fluctuation of the repulsive force which is obtained by the residual of the linear contact line fitting in Fig. 2b. The deviation of the repulsive force is 0.67 nN. According to the error transfer relation $\left|\frac{\Delta f}{\bar{f}}\right| = 13\left|\frac{\Delta d}{\bar{d}}\right|$, where $f_{rep} = Ad^{-13}$, and $\bar{f}$ is the mean repulsive force (11.05 nN), and $\bar{d}$ is the mean distance after vdW contact (0.3 nm), the deviation of the tip-sample distance after vdW contact is 0.0014 nm.

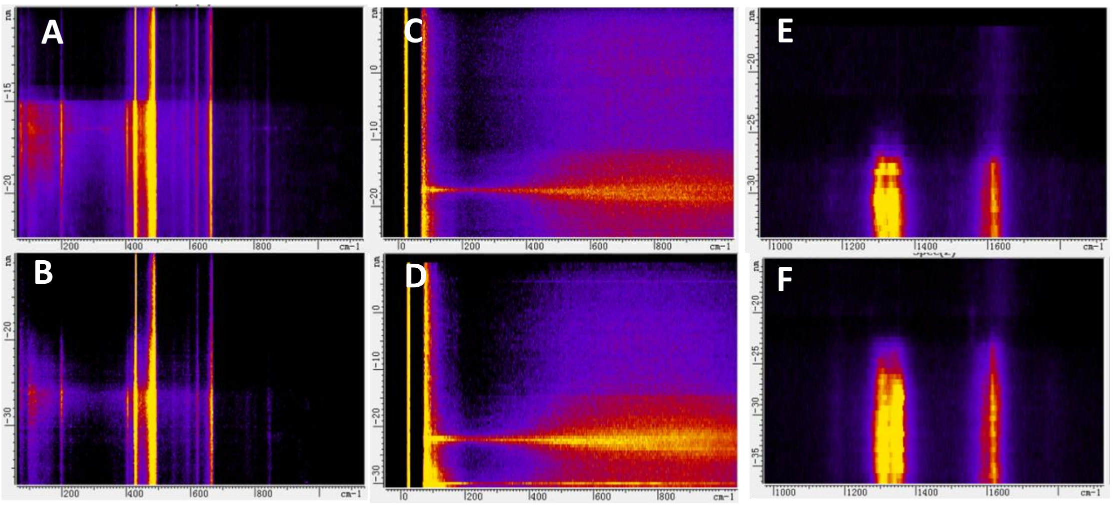

Supplementary Figure 5. **Reproducibility of TERS measurements.** Similar results were obtained from several spots in FL-MoS_2 flake (**a** and **b**), on gold substrate (**c** and **d**) and on carbon nanotubes (**e** and **f**).

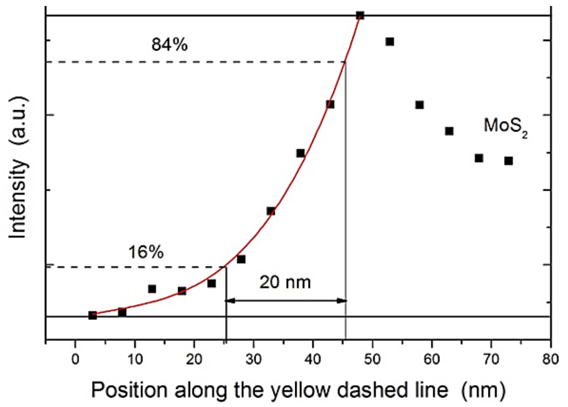

Supplementary Figure 6. Spatial resolution of optical imaging. Optical profile along the yellow dashed line in Fig. 4a provides an upper bound for spatial resolution of ~ 20 nm for optical imaging.

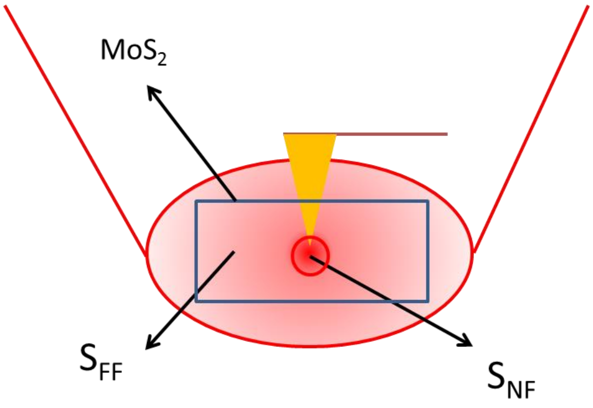

Supplementary Figure 7. Schematic of the incident and local fields for enhancement factor (EF) calculations. Large red ellipse represents the focused incident laser electric field which covers the FL-MoS_2 flake shown by the blue rectangle. The area of the flake was estimated from the AFM image in Fig. 1c to be S_{FF} = 200*800 nm = 160000 nm^2 and was used as the far-field normalization factor in the EF equation [8]:

$$EF = \left(\frac{FF + NF}{FF} - 1 \right) \frac{S_{FF}}{S_{NF}}$$

Here, the numerator FF + NF and the denominator FF are the intensities of the optical signals with the gold tip in contact and out of contact with the FL-MoS_2, respectively. The near field normalization factor $S_{NF} = \pi(R_{NF})^2$ = 1256 nm^2 was estimated by assuming the area of the near field spot to be approximately equal to the size of the tip with the radius R_{NF} = 20 nm. The calculated EFs for selected strongest Raman transitions of the FL-MoS_2 flake in Fig. 1 with 0.32 nm gap are shown in Supplementary Table 1.

Supplementary Table 1. Enhancement factors (EFs) for selected strongest Raman transitions of the FL-MoS_2 flake in Fig. 1 with 0.32 nm gap estimated as shown in Supplementary Fig. 7.

**Transition (cm^{-1})**	**EF**
A_{1g}-LA (177)	124
E_{2g}^1 (382)	284
A_{1g} (408)	3
A_{2u} (464)	58
E_{2g}^1+LA (599)	-6
A_{1g}+LA (642)	46

Supplementary Note 1

Tip-sample distance dependence analysis

The AFM cantilever can be regarded as a spring with the force - deflection dependence given by Hooke's law: $F = k\delta_c$, where δ_c is the cantilever deflection. The spring constant of the cantilever used in this work was $k = 2.8N/m$. During the measurements, the tip was kept stationary and the sample moved upwards in discrete steps (0.2 nm), driven by the piezoelectric actuator with the precision of ~ 0.1 nm. The tip-sample piezo displacement and the corresponding cantilever deflection were recorded and used to calculate the actual tip-sample distance by following the procedure summarized in Fig. 2 and described in more detail below. Supplementary Fig. 2 shows the plot of the cantilever deflection versus the tip-sample piezo displacement. We denote four typical moments to illustrate the behavior of the tip during the tip-sample approach. During moment (1), the sample is far from the tip, and the interaction force between the tip and the sample is negligible. Therefore, the cantilever deflection stays constant. At moment (2), the displacement-deflection curve reveals a singularity, which means that the cantilever is dragged and "jumps" onto the sample. This is the so-called "Snap to contact".[1] In the high vacuum and low temperature conditions, the "Snap to contact" occurs typically at the subnanometer length scale of tip-sample separation, when the derivative of the force versus tip-sample separation exceeds the spring constant of the cantilever. However, in ambient environment, a liquid film (vapor) can be adsorbed on the sample surface.[2, 3] The enhanced adhesive force of the vapor film gives rise to a sudden "jump" over ~ 2 nm, when the tip is at the position of ~ 5 nm above the sample surface. After the contact, the tip penetrates the film which can only exert a limited repulsive force, while the attractive force is still dominant. Therefore, the cantilever deflection slightly decreases.[4] At moment (3), the tip breaks through the film and contacts the sample surface. The red line in Supplementary Fig. 2 denotes the so-called "contact line," which shows the linear coupling of the sample and the tip movements.[3, 5] We

assumed that at the vdW contact the repulsive force is 0.5 nN, which corresponds to 3.26 A^o vdW diameter.

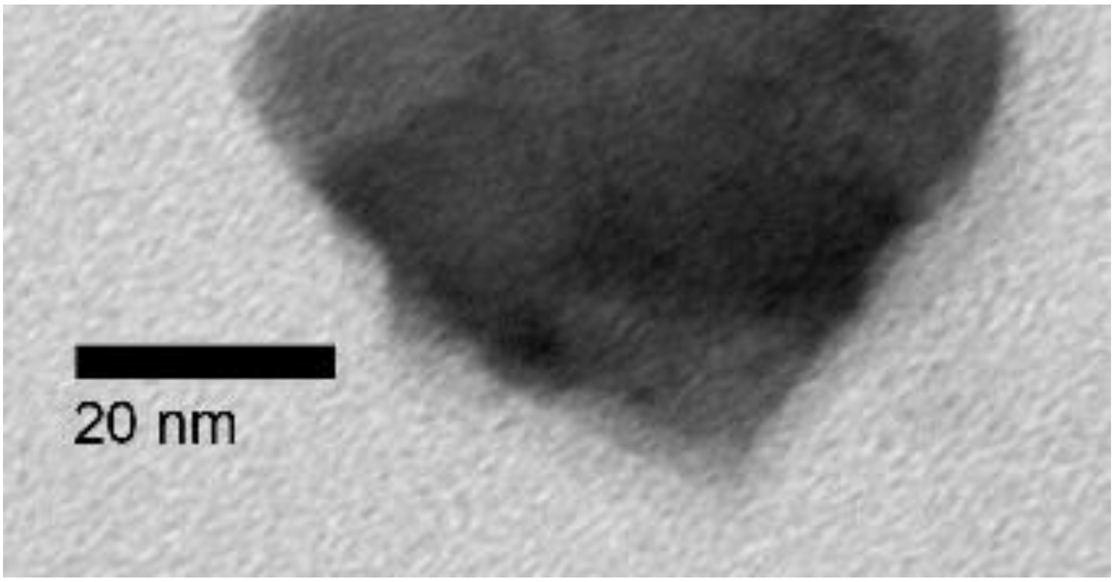

Supplementary Figure 8. Transmission electron microscopy (TEM) image of a typical few-layer MoS_2 flake.

Supplementary References

1. Cappella, B. and G. Dietler, *Force-distance curves by atomic force microscopy.* Surface science reports, 1999. **34**(1): p. 1-104.
2. Otto, A., *Surface-enhanced Raman scattering:"Classical" and "Chemical" origins*, in *Light scattering in solids IV*. 1984, Springer. p. 289-418.
3. Weisenhorn, A., et al., *Measuring adhesion, attraction, and repulsion between surfaces in liquids with an atomic-force microscope.* Physical Review B, 1992. **45**(19): p. 11226.
4. Mate, C.M. and V. Novotny, *Molecular conformation and disjoining pressure of polymeric liquid films.* The Journal of chemical physics, 1991. **94**(12): p. 8420-8427.
5. Hoh, J.H. and A. Engel, *Friction effects on force measurements with an atomic force microscope.* Langmuir, 1993. **9**(11): p. 3310-3312.
6. Kycia, A.H., et al., *Atomic force microscopy studies of a floating-bilayer lipid membrane on a Au (111) surface modified with a hydrophilic monolayer.* Langmuir, 2011. **27**(17): p. 10867-10877.

7. Garcia-Manyes, S. and F. Sanz, *Nanomechanics of lipid bilayers by force spectroscopy with AFM: a perspective.* Biochimica et Biophysica Acta (BBA)-Biomembranes, 2010. **1798**(4): p. 741-749.
8. Pettinger, B., et al., *Tip-enhanced Raman spectroscopy: near-fields acting on a few molecules.* Annual review of physical chemistry, 2012. **63**: p. 379-399.
